# Multi-scale cortical bone traits vary in females and males from two mouse models of genetic diversity

**DOI:** 10.1093/jbmrpl/ziae019

**Published:** 2024-02-21

**Authors:** Nicole Migotsky, Surabhi Kumar, John T Shuster, Jennifer C Coulombe, Bhavya Senwar, Adrian A Gestos, Charles R Farber, Virginia L Ferguson, Matthew J Silva

**Affiliations:** Orthopaedic Surgery, Washington University in St. Louis, St. Louis, MO 63110, United States; Department of Biomedical Engineering, Washington University in St. Louis, St. Louis, MO 63110, United States; Orthopaedic Surgery, Washington University in St. Louis, St. Louis, MO 63110, United States; Orthopaedic Surgery, Washington University in St. Louis, St. Louis, MO 63110, United States; Department of Mechanical Engineering, University of Colorado, Boulder, CO 80309, United States; Department of Mechanical Engineering, University of Colorado, Boulder, CO 80309, United States; Materials Instrumentation and Multimodal Imaging Core, University of Colorado, Boulder, CO 80309, United States; Center for Public Health Genomics, University of Virginia, Charlottesville, VA 22908, United States; Department of Mechanical Engineering, University of Colorado, Boulder, CO 80309, United States; Materials Instrumentation and Multimodal Imaging Core, University of Colorado, Boulder, CO 80309, United States; Orthopaedic Surgery, Washington University in St. Louis, St. Louis, MO 63110, United States; Department of Biomedical Engineering, Washington University in St. Louis, St. Louis, MO 63110, United States

**Keywords:** bone microCT, osteocytes, genetic research, biomechanics, bone matrix

## Abstract

Understanding the genetic basis of cortical bone traits can allow for the discovery of novel genes or biological pathways regulating bone health. Mice are the most widely used mammalian model for skeletal biology and allow for the quantification of traits that cannot easily be evaluated in humans, such as osteocyte lacunar morphology. The goal of our study was to investigate the effect of genetic diversity on multi-scale cortical bone traits of 3 long bones in skeletally-mature mice. We measured bone morphology, mechanical properties, material properties, lacunar morphology, and mineral composition of mouse bones from 2 populations of genetic diversity. Additionally, we compared how intrabone relationships varied in the 2 populations. Our first population of genetic diversity included 72 females and 72 males from the 8 inbred founder strains used to create the Diversity Outbred (DO) population. These 8 strains together span almost 90% of the genetic diversity found in mice (*Mus musculus*). Our second population of genetic diversity included 25 genetically unique, outbred females and 25 males from the DO population. We show that multi-scale cortical bone traits vary significantly with genetic background; heritability values range from 21% to 99% indicating genetic control of bone traits across length scales. We show for the first time that lacunar shape and number are highly heritable. Comparing the 2 populations of genetic diversity, we show that each DO mouse does not resemble a single inbred founder, but instead the outbred mice display hybrid phenotypes with the elimination of extreme values. Additionally, intrabone relationships (eg, ultimate force vs. cortical area) were mainly conserved in our 2 populations. Overall, this work supports future use of these genetically diverse populations to discover novel genes contributing to cortical bone traits, especially at the lacunar length scale.

## Graphical Abstract

**Graphical Abstract:**
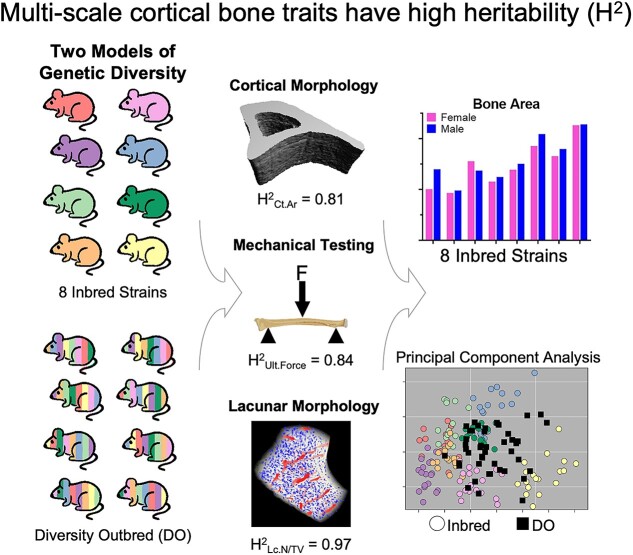

## Introduction

Osteoporosis affects more than 54 million people in the USA and contributes to increased fracture risk.^1–3^ About 70% of variability in bone mineral density (BMD) and 60% of variability in osteoporotic fracture risk are genetically based, ie, heritable.^4–6^ Many traits in addition to BMD contribute to bone strength and fracture risk, such as morphology, mineralization, and stiffness. Although Karasik et al. reported heritability of bone morphology as high as 98%,^7^ data on the heritability of most skeletal traits in humans are lacking.

Due to the high homology between mammalian genomes, results from mice can assist in understanding the complicated effects of genetics in humans.^8^ Environmental factors can be well controlled in mice and complex phenotypes can be measured (eg, bone strength). A recent study showed that all 55 skeletal phenotypes measured in 12-wk-old genetically diverse mice had non-zero heritability.^9^ Understanding the genetic basis of bone traits can lead to the discovery of novel genes or pathways as therapeutic targets for osteoporosis.^10^

There remain gaps in the literature regarding the heritability of bone traits. First, almost 80% of genome-wide association study (GWAS) participants are of European descent.^11^ Likewise, the reported heritability of human bone morphology is based on subjects of European descent.^7^ Second, previous mouse studies only investigated a single long bone, primarily the femur.^9^^,^^12^^,^^13^ Many bone traits are site-dependent, so heritability may vary between long bones. Third, measured traits focused on the whole bone or tissue length scale. The osteocyte is the most abundant cell type in bone and is responsible for regulating bone remodeling to maintain bone health. Therefore, it is important to understand bone traits at the cellular length scale, such as the shape and density of osteocytes. Finally, heritability estimates for bone traits were previously done using skeletally-developing mice (11–13 wk old) or elderly humans (avg 72 yr old), leaving a gap to investigate the heritably of traits in the young-adult, skeletally mature skeleton. Bone mass and fracture risk later in life depend on the peak bone mass attained as a young-adult,^14^ making it important to study factors that affect the young-adult skeleton.

To facilitate studies of the genetic basis of complex diseases and phenotypes, The Jackson Laboratory (JAX) generated the Diversity Outbred (DO) mouse population by cross-breeding 8 inbred founder strains to produce a population with random assortments of alleles, thus modeling the heterozygosity and phenotypic diversity of the human population.^15^^,^^16^ These 8 inbred strains consist of 3 wild-derived strains (CAST/EiJ, PWK/PhJ, and WSB/EiJ) and 5 classical laboratory strains (A/J, C57BL/6 J, 129S1/SvImJ, NOD/ShiLtJ, and NZO/HlLtJ) that together cover ~90% of the genetic diversity in the mouse genome and represent the 3 subspecies of the common house mouse (*Mus musculus domesticus*, *Mus musculus musculus*, and *Mus musculus castaneous*).^17^ To date, these genetically diverse populations have been used to evaluate the heritability and candidate genes regulating cancellous bone microarchitecture in the growing skeleton,^13^ the bone response to hindlimb disuse,^12^ and the heritability of femur properties and cortical bone accrual in the growing skeleton.^9^ These studies provide rationale to use these populations, both the inbred founders and DO mice, to further study the heritability of cortical bone traits at different length scales.

We aimed to calculate the heritability of multi-scale cortical bone traits of 3 long bones (radius, tibia, and femur) in skeletally mature female and male mice. We used cohorts of mice from 2 models of genetic diversity, each containing the same pool of alleles: (1) the 8 inbred founder strains used to create the DO population, and (2) the DO population. Comparing individual inbred strains, we hypothesized that cortical bone traits vary with genetic background and sex. Comparing traits between inbred founder and DO cohorts, we hypothesized that the DO cohort has a similar spread and mean per trait as the inbred founders. Comparing correlations between traits within a single animal, we hypothesized that relationships between cortical bone traits are conserved in these 2 models.

## Materials and methods

### Mouse populations

**Figure 1:**
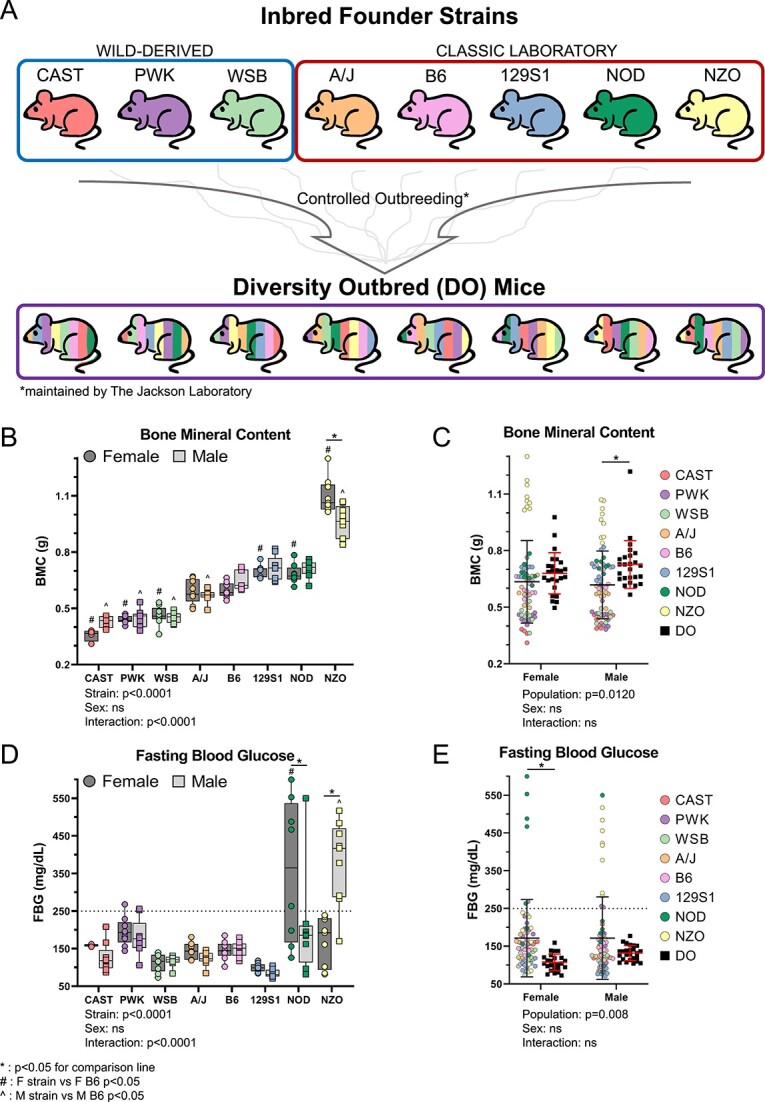
(A) Phenotyping was performed on 5-mo-old females and males from 2 genetically diverse mouse populations. (B) Whole body BMC of inbred founders. Strains on the *x*-axis are ordered from smallest to largest BMC. This order is maintained for all subsequent graphs. (C) BMC for DO mice compared to pooled inbred founder mice. (D) FBG of each inbred founder strain, and (E) pooled inbred founders and DO mice. Hyperglycemia defined as FBG > 250 mg/dL M. ^*^P <0.05 for comparison line; ^#^*P* <0.05, F strain vs F B6; ^^^*P* <0.05, M strain vs M B6.

This study was approved by the Washington University IACUC. Female and male mice from 2 populations were ordered from JAX: (1) Inbred mice from 8 strains (CAST/EiJ—JAX stock #000928, PWK/PhJ—#003715, WSB/EiJ—#001145, A/J—#000646, C57BL/6 J—#000664, 129S1/SvImJ—#002448, NOD/ShiLtJ—#001976, and NZO/HlLtJ—#002105), and (2) DO mice (#009376) generated by continuous outbreeding of the founder strains (Figure 1A). Inbred mice (*n* = 9/strain/sex) were delivered at 8 wk age and aged to 22 wk. Of the *N* = 144 that arrived, 8 died prematurely (3 NOD, 5 CAST) leaving *N* = 136 for analysis. DO mice (*n* = 25/sex, generations G46 and G47) were delivered between 3 and 5 wk and aged to 22 wk. One female DO mouse died prematurely, leaving 24 for analysis. Inbred mice were group-housed up to 5 per cage. Male DO mice were housed individually, and female DO mice were housed up to 3 per cage per JAX recommendations. Mice were kept on a 12-h light–dark schedule with ad libitum access to food and water.

### Longitudinal measurements

Inbred founder strains were monitored longitudinally for whole body traits. Mouse weights, dual-energy X-ray absorptiometry (DEXA) scans, and fasting blood glucose (FBG) were collected at 9, 12, 15, 18, and 22 wk old. FBG was of interest because NOD and NZO strains are susceptible to diabetes.^18^^,^^19^ Mice were fasted for 6 h, weighed, and FBG was measured using a drop of tail blood (Glucocard Vital, Arkray Inc.); hyperglycemia was defined as >250 mg/dL. For DEXA, mice were fully sedated (isoflurane 1.5%–4%) and scanned for whole body (excluding the head) BMC and areal bone mineral density (UltraFocus100, Faxitron, 4 × 40 kV and 6 × 80 kV scans). At 22 wk old, inbred mice were euthanized, bones collected for analysis (Table 1), and bodies stored frozen at −20°C.

**Table 1:** Bones and outcomes for inbred founders and diversity outbred (DO) mice.

**Bone**	**Outcome**	**Mice**
Right radius	μCT, 3 pt bend	Inbred founders, DO
Right tibia	μCT (Supp)	Inbred founders, DO
Left femur	μCT (Supp)	Inbred founders, DO
Left tibia	XRM	Inbred founders
Right femur	Raman	Inbred founders

DO mice were evaluated *in vivo* at 22 wk. Mice were fasted for 6 h, weighed, and FBG was measured. The next day mice were anesthetized with isoflurane for DEXA and subsequent tibial microCT (μCT) (*see Cortical Bone Morphology*). DO mice were subjected to right limb tibial loading (findings not reported here), euthanized at 25 wk age, femora and radii collected for analysis (Table 1), and bodies stored at −20°C. For tibial μCT, only scans from before loading in the DO mice were analyzed in this study. Inbred founder mice were not loaded.

### Cortical bone morphology

The right tibia was μCT scanned (vivaCT 40, Scanco Medical-70kVP, 8 W, 300 ms integration time) at 10.5 μm/pixel resolution either *ex vivo* (inbred founders) or *in vivo* (DO mice). A 1.05 mm long region (100 slices) centered 5 mm proximal to the distal tibiofibular junction (TFJ) was analyzed. The right radius and left femur were scanned (μCT50, Scanco Medical-70kVP, 4 W, 700 ms integration time) at 7.4 μm/pixel resolution ex vivo. A 1.48 mm long region (200 slices) centered at the radius midpoint was analyzed. A 1.11 mm long region (150 slices) centered at the femur midpoint was analyzed. Scans were analyzed following published guidelines^20^ for total area (Tt.Ar), bone area (Ct.Ar), medullary area (Ma.Ar), cortical thickness (Ct.Th), polar moment of inertia (pMOI), and tissue mineral density (TMD).

### Mechanical testing

The right radius was cleaned and stored at −20°C in PBS-soaked gauze. The radius was selected for testing because its relatively long, slender shape allows for more accurate estimation of material properties than the femur.^21^^,^^22^ Prior to testing, samples were brought to room temperature and kept hydrated in PBS. Bones were tested in 3-point bending with a bottom span of 7 mm, and the top point aligned with the bone center and preloaded to −0.1 N (Instron 8841). Bones were preconditioned for 5 cycles (−0.4 N amplitude, 1 Hz) then loaded monotonically to failure at 0.1 mm/s. Force–displacement curves were analyzed for stiffness (K), ultimate force (F_u_), yield force (F_y_), post-yield displacement (PYD), and work-to-fracture. Material properties (ultimate stress [S_u_], yield stress [S_y_], elastic modulus [E]) were estimated using engineering beam theory.^23^

### Osteocyte lacunar morphology

Osteocyte lacunar morphology was quantified in the inbred founder population. Left tibiae were dissected and fixed in 4% PFA overnight, then rinsed and stored in PBS. A region 4 mm proximal to the TFJ was scanned on an X-ray microscope (XRM, Xradia Versa 520, Zeiss). A prescan, using the 4× objective, was acquired to locate the high-resolution scan field-of-view (FOV), centered halfway between endocortical and periosteal surfaces at the postero-lateral apex (Supplementary Figure S8A). Scanning parameters for the high-resolution scan were: 40 kV, 3 W, 1601 projections, 20× objective, bin = 2, 4800–5000 projection intensity (7–8 s exposure), yielding a nominal resolution of 0.54 μm/voxel. Images were segmented, filtered, and analyzed using custom scripts in Dragonfly (version 4.1, Object Research Systems) for total lacunar volume, total vessel volume, and individual lacunar properties (volume, aspect ratio, phi, and sphericity) as described.^24^ More than 4000 individual lacunae were analyzed per bone.

### Raman spectroscopy

Cortical bone matrix composition was quantified in the inbred founder population. Right femurs were dehydrated in ethanol and embedded in (poly)methylmethacrylate (PMMA) (Thermo-Fisher Scientific AAA130300F). Plastic blocks were cut at the midpoint perpendicular to the bone long-axis and trimmed to 5 mm length. Block faces were polished to 0.05 μm (600 and 1200 grit silicon carbide sandpaper, 1 μm and 0.05 μm aluminum oxide) on Rayon felt pads (Allied). Measurements were taken on the anterior side of the femur in lamellar bone, excluding periosteal and endocortical surfaces (Supplementary Figure S1). Measurements were taken in a grid of 3 spots wide spanning the cortical width (5–9 spots long) spaced 20 μm apart. Spectra were collected using a red laser (785 nm wavelength) on a Renishaw inVia confocal microscopy system. Each point was collected with 10 accumulations with a 6-s exposure time and post-processed using WiRE 4.1 software (baseline subtraction with 11th order polynomial fit, cosmic ray removal, spectra normalization). A single measurement of the PMMA for each sample was used for baseline subtraction. Data were analyzed in a custom MATLAB code to quantify area ratios of mineral:matrix (v2 phosphate: amide III, v1 phosphate:proline), carbonate:phosphate (v1 carbonate: v1 phosphate), and crystallinity (inverse of full-width at half-maximum of v1 phosphate).^25–27^

### Correlation matrix

Matrices of Pearson’s correlation coefficients were computed and plotted using R (v4.0.2, functions *cor* and *corrplot*) on 3 separate datasets: radial and whole body traits in (1) inbred founders, (2) DO mice, and (3) lacunar traits in inbred founders. For radial traits, variables were ordered by measurement technique consistently for each dataset. For lacunar traits, variables were clustered into 3 groups using the *ward.D2* unbiased, hierarchical algorithm.

### Principal component analysis

Principal component analysis (PCA) is a method for dimensional reduction that can facilitate between-group comparisons in studies that assess numerous bone traits.^22^^,^^28^^,^^29^ PCA was performed using R on 2 datasets from the inbred founder population: (1) 15 radial and whole body traits, and (2) 10 lacunar traits. Each trait was centered and scaled within the *prcomp* function (mean = 0, SD = 1), and its contribution to each principal component (PC) was determined. For visualization of the inbred founders, data points were grouped by mouse strain and encompassed by a normal data ellipse spanning 1 SD. Next, to compare inbred founder and DO populations for dataset 1 (radial and whole body traits), each DO animal was plotted onto the PCA space defined by the inbred founders. The value for each DO trait was centered and scaled and linearly combined according to PC weightings from the inbred founders.

### Heritability calculations

Broad-sense heritability (H^2^) was calculated for all traits measured in inbred founders as the proportion of variance due to genetic differences^30^: H^2^ = σ^2^_strain_/(σ^2^_strain_ + σ^2^_sex_ + σ^2^_res_), where σ^2^_strain_ is the between-strain variance, σ^2^_sex_ is the between-sex variance, and σ^2^_res_ is the residual variance (Table 2). Variances were calculated from the sum of squares from a 2-factor ANOVA with strain and sex as factors: σ^2^_strain_ = SS_strain_/n_avg_ (n_avg_ = average group size), σ^2^_sex_ = SS_sex_/df_sex_, and σ^2^_res_ = SS_res_/df_res_.

**Table 2:** Broad sense heritability was calculated using data for the inbred founders, defined as the fraction of total variation due to strain.

**Trait**	**Heritability (H** ^**2**^ **)**	**Trait**	**Heritability (H** ^**2**^ **)**	
BMC	0.993	Lc.AspectRatio	0.755	
Cortical thickness	0.985	Ult. Stress	0.746	
TMD	0.967	Work-to-Fx	0.741	
Lc.No Density	0.966	Medullary area	0.722	Broad Sense heritability
FBG	0.91	Yield force	0.718	${\sigma}_{strain}^2=\frac{S{S}_{strain}}{n}$
Lc.Sphericity	0.887	Yield stress	0.717	${\sigma}_{sex}^2=\frac{S{S}_{sex}}{d{f}_{sex}}$
Cortical area	0.884	Porosity	0.689	${\sigma}_{res}^2=\frac{S{S}_{res}}{d{f}_{res}}$
Weight	0.84	Modulus (E)	0.684	${H}_{broad}^2=\frac{\sigma_{strain}^2}{\sigma_{strain}^2+{\sigma}_{sex}^2+{\sigma}_{res}^2}$
Ult. Force	0.836	Lc.MaxFeret	0.634	
Lc.SD.Phi	0.835	Lc.Vol/SA	0.535	
V.Vol Density	0.827	Lc.Vol Density	0.516	
pMOI	0.799	Lc.Vol	0.383	
Total area	0.799	PYD	0.209	
Stiffness (K)	0.776			

### Statistical analysis

Statistical analysis was done in GraphPad Prism (v9). Differences between the 8 inbred founders were determined using 2-factor ANOVA (factors: mouse strain, sex). Comparisons between the inbred founder population (all 8 strains pooled) and the DO population were made using 2-factor ANOVA (factors: population, sex). Post hoc tests were run with Sidak correction. Significance was set at *P* < .05. Body mass was evaluated as a co-variate using a general linear model in SPSS (IBM). Body-mass adjusted values per animal were calculated according as described^23^: ${Trait}_{adjusted}={Trait}_{unadjusted}- slope\ast \left( body\ mass- mean\ body\ mass\right)$. Slopes were determined by linear regression of each trait with body mass within each strain group (8 groups). To ensure statistically meaningful relationships, for any regression with a *P* >0 .20, the slope was set to 0 for that strain. The mean body mass was calculated as the mean of the means of each strain group (mean of 8 means).

## Results

### Young-adult mice from inbred founder and DO populations span a large range of body size and bone mass

At the whole body length scale, DEXA was used to assess skeletal mass. The 8 inbred founder strains were skeletally mature by 22 wk; the average increase in BMC between 18 and 22 wk is less than 4% (Supplementary Figure S2A). At 22 wk, BMC of inbred founders varied significantly between strains, with a 3-fold difference between CAST females and NZO females (Figure 1B; strains are ordered from smallest to largest BMC along the *x*-axis for all graphs in this report). The DO population had significantly higher BMC than inbred founders with a narrower range of values and higher minima (Figure 1C). About 12% of inbred founders were hyperglycemic by 22 wk of age (Figure 1D). NZO males became hyperglycemic between 12 and 15 wk, while NOD females became hyperglycemic between 18 and 22 wk (Supplementary Figure S2). NOD females and NZO males have large variations in FBG glucose likely due to varied timing of diabetic onset. In contrast, no DO mice were hyperglycemic at 22 wk (Figure 1E).

### Cortical morphology of 3 long bones varies strongly with mouse strain and modestly with sex

**Figure 2:**
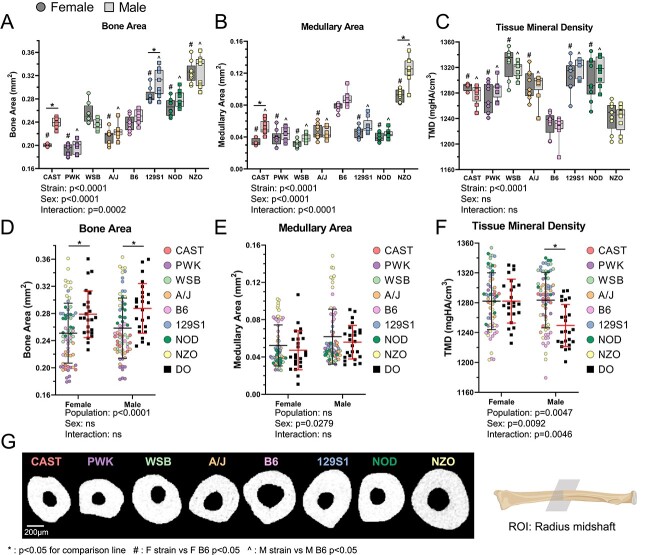
Radial morphology varies with strain in a sex-dependent manner. (A) Bone area, (B) medullary area, and (C) TMD of inbred founder mice. (D) Bone area, (E) medullary area, and (F) TMD of inbred founder and DO populations. (G) Representative cross-sections at the radius mid-diaphysis show the variation in bone shape and size between the 8 inbred founder strains (females). ^*^P<0.05 for comparison line; ^#^*P*<0.05, F strain vs F B6; ^^^*P*<0.05, M strain vs M B6

Whole bone morphology of the radius, assessed using μCT, varied significantly between inbred founder strains in a sex-dependent manner (Figure 2, Supplementary Figure S3). Compared to inbred founders, the DO population on average had larger bones with a similar medullary area (Figure 2D and E). Analysis of morphology from tibias (Supplementary Figure S5) and femurs (Supplementary Figure S6) showed similar population spreads and differences between strains as observed for the radius, indicating consistent strain effects across long bones. Variance in morphology was due much more to mouse strain (genetics) than sex. For cortical bone area, over 80% of total variation in inbred founders was due to strain (radius: 88%, tibia: 87%, femur: 82%), while only about 2% was due to sex (radius: 1.5%, tibia: 2.3%, femur: 1.8%). Radius TMD also varied significantly between inbred founder strains, independent of sex (Figure 2C). The DO males, on average, had lower TMD than inbred founder males, but no difference was observed between females (Figure 2F). In summary, cortical morphology varied widely between inbred founder strains with outbred animals (DO) having larger bones on average, whereas differences between sexes were modest and strain-dependent.

### Cortical bone mechanical properties vary with mouse strain and sex, but material properties only vary with mouse strain

**Figure 3:**
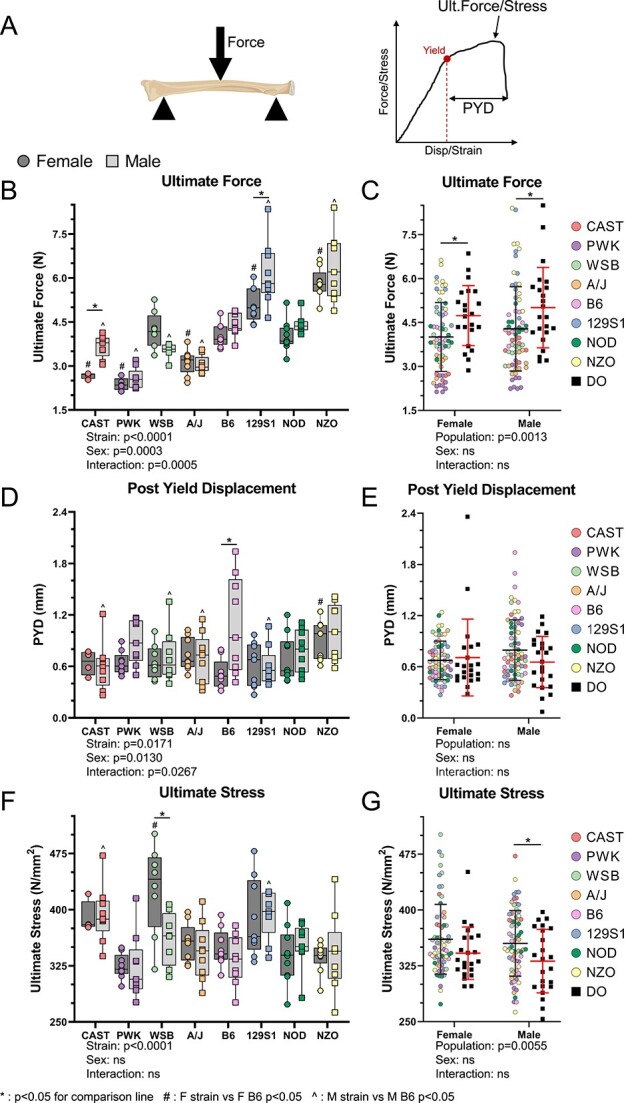
Radius mechanical and material properties vary between inbred mouse strains. (A)The radius was tested using 3-point bending to determine mechanical properties. Beam theory equations were used to estimate material properties. (B) Ultimate force of the inbred founder strains individually, and (C) pooled inbred founder compared to the DO population. (D) PYD (a measure of ductility) of the inbred founder strains, and (E) the inbred founders compared to the DO population. (F) Ultimate stress across the inbred founder mice, and (G) the inbred founders compared to the DO population. ^*^P<0.05 for comparison line; ^#^*P*<0.05, F strain vs F B6; ^^^*P*<0.05, M strain vs M B6

The radius was tested in 3-point bending to determine whole bone mechanical and estimated material properties (Figure 3A).^21^ Mechanical properties varied between inbred founder mice, with significant strain, sex, and strain-sex interaction effects (Figure 3B and D and Supplementary Figure S4A–C). Mouse strain accounted for 80% of the total variation in ultimate force, while sex accounted for <2% of the variation (ANOVA). Compared to inbred founders, the DO population had stronger bones on average (Figure 3C). PYD, a measurement of ductility, varied only modestly with strain (Figure 3D). PYD was highly variable within each strain/sex group and mouse strain only accounted for about 11% of the total variation. PYD did not differ between inbred founder and DO populations (Figure 3E). All estimated material properties varied between inbred founder strains but did not differ between sexes. The DO population had slightly lower ultimate stress and other material properties compared to the inbred founder population (Figure 3G; Supplementary Figure S4I and J). In summary, radial bone mechanical and material properties vary between inbred founder strains, with outbred mice having on average higher mechanical but lower material properties than inbred founders.

### Within-bone correlations are similar in both mouse populations, but stronger in inbred mice

**Figure 4:**
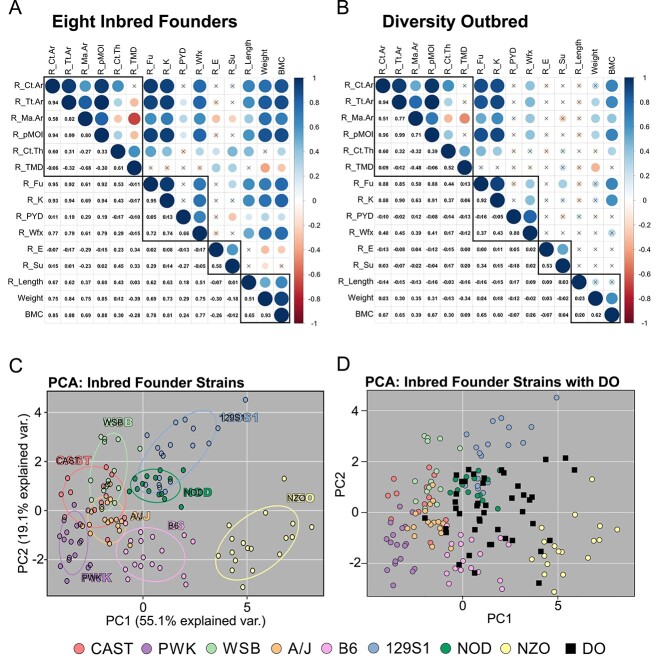
Matrix of Pearson’s correlations between traits measured in the radius of the (A) 8 inbred founder strains, and (B) DO mice. Black boxes separate different types of outcomes (morphology, mechanical properties, material properties, whole skeleton size). ‘X’ indicates a nonsignificant correlation (*P* > 0.05). (C) PCA analysis showing how inbred founders cluster by strain. (D) DO mice were mapped onto the PCA space defined by the inbred founders.

In inbred founders, bone size traits (bone area, total area, medullary area, and pMOI) were all highly correlated (Figure 4A). Cortical thickness was only moderately correlated with other morphology parameters. Size traits were highly correlated with whole bone mechanical properties (except PYD) as well whole body measurements (bone length, weight, BMC). Mechanical properties (except PYD) were also correlated with whole body measurements. Ultimate force, an important trait for bone function, correlates highly with bone morphology, bone stiffness, and BMC in inbred founders. Bone material properties correlated with each other, but did not correlate with size parameters, which is expected because material properties are already normalized for bone size. TMD had no strong correlates, but was moderately correlated with cortical thickness (*r* = 0.61) and medullary area (*r* = −0.68).

Compared to inbred founders, the DO population showed similar correlations between traits, but the magnitudes were weaker. In particular, correlations with body weight and length were much weaker in DO mice (Figure 4B). The correlation between total area and weight was lower by 0.54 (0.84 in the Founder vs 0.30 in the DO). On the other hand, the relationship between ultimate force and bone area was highly, positively correlated (*r* = 0.95 in inbred founders and 0.88 in DO) and conserved in the 2 populations, sharing the same slope and intercept (Supplementary Figure S9).

### Inbred founder strains separate using PCA while DO mice overlap many individual strains and occupy gaps between strains

To complement the analyses of individual traits, we used PCA to examine how these traits collectively contributed to differences within and between strains. Based on PCA of 15 radial traits in inbred founders, the first 2 PCs explained almost 75% of the population variance, and mice strongly clustered by strain (Figure 4C). Mouse size, bone size, and bone strength parameters (BMC, weight, Ct.Ar, Tt.Ar, pMOI, F_u_, K) were the main contributors to PC1. Bone thickness and material properties (Ct.Th, TMD, S_u_, E) were the main contributors to PC2 (Supplementary Figure S7C). NZO was distinct from all other strains, while B6 was mostly distinct. The wild-derived strains showed moderate overlap with each other (CAST overlaps WSB and PWK) and with A/J. When the DO mice were mapped onto the PCA space defined by the inbred founders, they occupy a smaller, central region of the space, eliminating extreme values at the periphery (Figure 4D). Additionally, many DO mice filled the empty space between the NZO and 129S1 clusters, indicating that individual DO mice have a unique combination of bone traits different from any single inbred founder mouse.

### Osteocyte lacunar morphology varies between inbred founder strains

**Figure 5:**
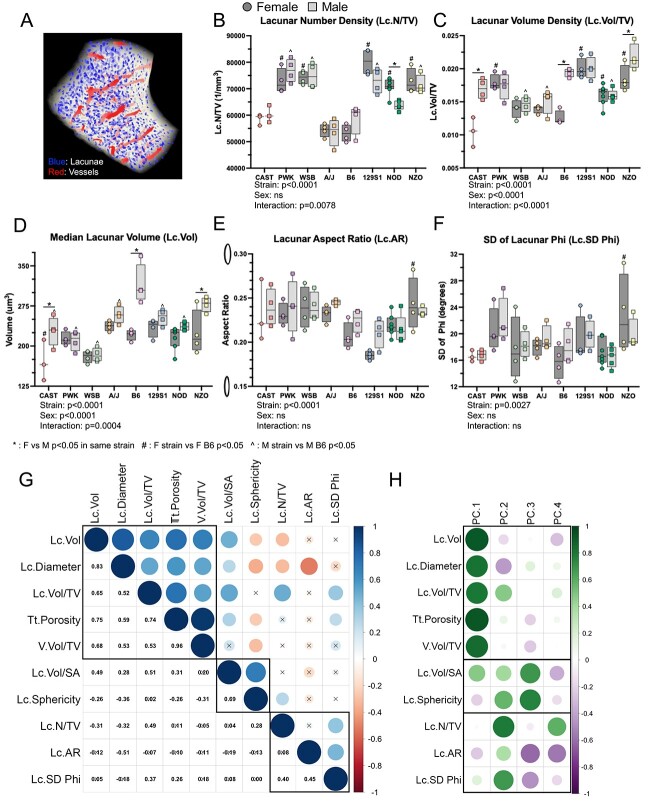
Osteocyte lacunar morphology varies between inbred strains. (A) Lacunae were imaged using X-ray microscopy (XRM). Representative section in the FOV, showing lacunae (blue) and vasculature (red). (B) Lacunar number density and (C) lacunar volume density. (D) The median lacunar volume. (E) The aspect ratio of each lacuna was analyzed as a measure of elongation; a ratio of 0 corresponds to a straight line, while 1 is a circle. Ellipses shown along the *y*-axis represent aspect ratios of 0.15 and 0.30. (F) SD of Phi (angle from z-axis) indicates how uniformly aligned the lacunae are (smaller value = more aligned). (G) Pearson’s correlation matrix of lacunar traits measured in inbred founders. Black boxes group traits that cluster using hierarchical clustering. (H) Contributions of each lacunar trait to the first 4 PCs of PCA. ^*^P<0.05, F vs M in same strain; ^#^*P*<0.05, F strain vs F B6; ^^^*P*<0.05, M strain vs M B6 *P*<0.05

Osteocytes and their lacunae are the site of mineral turnover to maintain homeostasis, and therefore lacunar morphology may be an important aspect of bone health. Total lacunar number density (Lc.N/TV) varied significantly between strains in a sex-dependent manner ranging from 53 189/mm^3^ in B6 females to 80 200/mm^3^ in 129S1 females (Figure 5B). The total volume of lacunae (which accounts for only 1-2% of total bone volume) varied significantly between inbred founders (Figure 5C). The median volume of individual lacuna (Lc.Vol) also varied with strain (Figure 5D). Lacunar volume density (Lc.Vol/TV) was strongly correlated to median lacunar volume (*r* = 0.65), and moderately correlated with lacunar number density (*r* = 0.49), indicating that total lacunar volume is increased mainly by increasing the volume of individual lacuna rather than their number (Figure 5G).

Median lacunar aspect ratio (Lc.AR), a measure of elongation, varied significantly between inbred founder strains (Figure 5E). Uniformity of lacunar orientation within each bone was defined as SD of Phi (angle of the principal lacunar axis from the z-axis). Lc.SD Phi varied significantly with mouse strain (Figure 5F) and correlated significantly with both lacunar elongation (Lc.AR) and lacunar number density (Lc.N/TV) (Figure 5G). Thus, in bones where lacunae are more aligned to each other, they tend to be more elongated and less densely packed. The lacunar traits that cluster together also contributed similarly to explain the population variance (Figure 5H). In summary, all osteocyte lacunar morphology traits are dependent upon mouse strain.

### Bone matrix composition minimally varies between inbred founder mice

**Figure 6:**
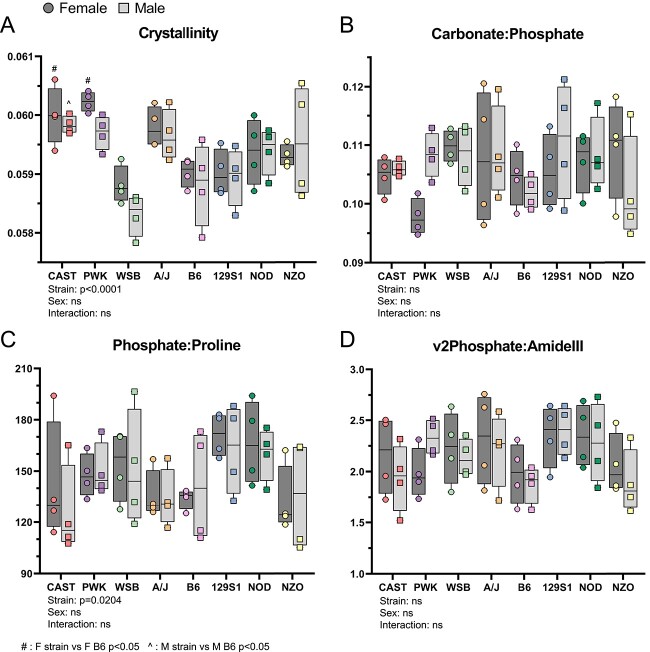
Raman spectroscopy was used to evaluate bone matrix composition of inbred founder femurs. (A) Crystallinity, (B) carbonate:phosphate, (C) Phosphate:Proline, and (D) v2phosphate:amideIII ratios. ^#^*P*<0.05, F strain vs F B6; ^^^*P*<0.05, M strain vs M B6 *P*<0.05

Material properties varied between inbred founders, suggesting that bone composition may vary with genetics. Therefore, bone composition was analyzed using Raman spectroscopy on the transverse cross-section of the femur midsection (Supplementary Figure S1). Crystallinity, a measure affected by mineral length and organization, varied significantly between mouse strains (Figure 6A). The carbonate:phosphate ratio, which varies with architecture, age, and crystallinity,^26^^,^^31^ did not vary between mouse strains (Figure 6B). One measurement of mineral:matrix (phosphate:proline) also varied significantly between mouse strains (Figure 6C). However, when mineral:matrix was measured using phosphate:amideIII, there was no significant variation (Figure 6D). In summary, the crystalline structure of the mineral varies modestly between inbred founders strains.

### Multi-scale cortical bone traits are moderately to highly heritable in the inbred founder population

Broad-sense heritability was calculated for traits measured in inbred founders (Table 2). BMC had the highest heritability (H^2^ = 0.993) indicating almost all the variance in the population is attributed to genetic differences. In general, lacunar traits had lower heritability, although there were a few exceptions, such as lacunar number density (H^2^ = 0.966). Heritability values were similar between long bones. For the radius, femur, and tibia, cortical thickness and cortical area were the most heritable morphology traits. Overall, of the 43 whole body, whole bone (radius, tibia, and femur), tissue, and lacunar traits, 35 (~80%) had a heritability above 60%, which is similar or greater than the reported heritability of BMD in humans.^4–6^

### The significant differences in bone traits between mouse strains are not due to differences in mouse size

Many bone traits correlated significantly with body weight (Figure 4A). Therefore, data were reanalyzed using ANCOVA with body mass as a covariate. Body mass was a significant covariate for 22 out of 42 traits measured. (Notably, no lacunar level trait had weight as a significant covariate.) Nonetheless, the significant effect due to mouse strain was maintained for 39 of 40 traits that were significant without body mass adjustment (Supplementary Table 1). PYD was the only trait that lost significance for strain after adjusting for body mass, although the significance of the strain–sex interaction was maintained. We also performed PCA with body weight adjusted traits, and observed no important changes in the findings (Supplementary Figure S7B–D). Likewise, heritability values were not meaningfully affected by body weight adjustment (Supplementary Tables 2 and 3). Therefore, although body weight is an important factor when considering differences in skeletal traits between animals, the significant effects of mouse strain in this study cannot be explained by differences in mouse size (body weight).

## Discussion

Using 2 mouse models of genetic diversity, we show that cortical bone traits in young-adult mice, from whole body to osteocyte-lacunar length scale, vary with genetic background and are heritable. In the radius, whole bone traits (morphology, mechanical properties) vary with both mouse strain (genetic background) and sex. Tissue level traits (TMD, material properties, bone composition) vary with genetic background but not sex. Comparing the 2 populations, the DO mice are on average bigger, are protected from hyperglycemia, and have larger, stronger bones. When all whole body and radial traits are evaluated together, the DO population occupies a subset of the PC space defined by the inbred founders. Many DO mice do not resemble any single inbred strain, implying that complex gene interactions determine skeletal traits. Overall, genetic background significantly contributes to cortical bone phenotype, which indicates genetic control of bone traits across length scales.

The findings support our first hypothesis that cortical bone traits vary with genetic background, consistent with a previous report that compared inbred mice.^32^ Of the 43 traits we measured in the inbred founder strains, only 2 (carbonate:phosphate and phosphate:amideIII ratios) did not vary significantly with strain. Additionally, broad-sense heritability (ie, the proportion of variability due to genetic difference), was greater than 20% for all traits, and after adjusting for body size, heritability was greater than 77% for all traits. These values are higher than those reported by Al-Barghouthi et al.,^9^ who reported heritability in DO mice as low as 12% after covariate adjustment. The mice used in that study were 12 wk old, and differences in rates of skeletal maturation may have been an added source of variation. Furthermore, we calculated heritability using data from the eight inbred founder strains, where each group has genetically identical replicates reducing the intrastrain variation. The greatest variation we found between strains was for morphology and mechanical properties (eg, cortical area, ultimate force). These traits tend to have higher heritability values, larger fold-differences between high and low groups, and contribute to the first principal component (PC1) of the PCA, which explains 55% of population variation. Al-Barghouthi et al. also found that cortical morphology traits had the highest heritability.^9^ In contrast, we observed that the tissue-level properties (eg, ultimate stress, TMD, mineral:matrix) tend to have less variability between strains and more variability within a strain. These traits have lower fold-differences and mainly contribute to PC2, which explains only about 20% of population variance. Overall, these results indicate that bone material properties are fairly conserved between strains, whereas how this bone is distributed varies markedly between strains.

To our knowledge, this is the first study to quantify osteocyte lacunar morphology across different mouse strains, extending previous work on mice from a C57BL/6 background.^33–35^ Lacunar size traits (eg, diameter, volume) tend to have moderate heritability (H^2^ = 0.38-0.63). By contrast, the number of lacunae (eg, lacunar number density) and their shape (eg, sphericity, aspect ratio) have high heritability (0.76-0.97). Therefore, the shape and number of lacunae are strongly determined by genetics in the inbred founder mice.

Contrary to our second hypothesis, many traits of the DO population have different mean values and smaller ranges than the inbred founder population. For 24 of 29 traits, there was a significant difference in means between populations. DO mice had larger skeletons (BMC), body weight, and healthier glucose levels than inbred founders. For the radius, tibia, and femur, the DO bones were larger (eg, greater cortical area) and had higher whole bone mechanical properties, but diminished material properties. The combination of higher total area but lower TMD seen in the DO population matches the preferred bone trait set established in Jepsen et al.^36^ and resulted in greater ultimate force. The range of values for each trait in DO mice was generally lower than in Founders, and extreme values were eliminated. The latter result implies epistasis in the inbred founder strain with the extreme phenotype, since disruption of the exact allele combination removes the extremes.

Although bone phenotypes for inbred founders^12^ and DO mice^9^ have been reported, they have not been directly compared. Turner et al.^37^ compared 2 inbred strains (B6, C3H) to multiple recombinant inbred lines (BXH RI) and showed that none of the measured traits grouped the RI strains into subsets resembling either of the 2 progenitors. Additionally, none of the RXH RI strains had ultimate force values approaching the high-strength C3H femurs, supporting epistasis of bone traits. In contrast, Jepsen et al.^36^ compared A/J and B6 inbred strains to multiple recombinant inbred lines (AXB/BXA RI) and showed many RI lines with femurs smaller than A/J and larger than B6. Herein, we found that almost all DO mice had phenotype values bounded by those measured in inbred founders. Additionally, when traits were reduced using PCA, the DO mice spanned between the clusters of inbred founder strains indicating that individual DO mice did not simply copy individual inbred founder mice. Depending on the alleles available and their specific combinations, bone phenotypes vary considerably, indicating that complicated interactions between genomic regions control skeletal phenotypes.

In support of our third hypothesis, many relationships between traits seen in inbred founder mice are maintained in DO mice, especially those between morphology and mechanical properties. For example, we observed an almost identical positive, linear relationships between ultimate force and cortical area in the 2 populations, suggesting that this relationship is highly conserved across *M. musculus*. On the other hand, correlations between most traits and body weight were greatly diminished. In inbred founder strains (as reported in other populations^38^), a relatively large mouse typically has a large bone, but in DO mice, this relationship is disrupted. For example, the correlation between cortical area and weight is nonsignificant in DO mice (*r* = 0.75, inbred founders; *r* = 0.23 DO). Jepsen et al. compared femurs from multiple RI lines and described relationships between bone morphology and tissue quality.^36^ Specifically, there was a positive correlation between TMD and cortical thickness in femurs, which we observed for radii in the current study. Thus, our data support the idea that there is a coordinated, biological regulation of the relative rates of periosteal and endocortical expansion (which determine cortical thickness) and TMD.^36^

Because many traits in inbred founders correlated significantly with body weight, we asked whether differences in traits between strains were due solely to differences in body size. After adding body weight as a covariate to the ANOVA analysis, all traits maintained a significant strain or strain/sex effect. Additionally, the clustering of inbred strains by PCA became more distinct after body weight adjustment, and heritability increased for most traits. Therefore, variations in body size alone do not explain variations in bone traits, indicating that there are genes that independently control bone traits and body size.

We acknowledge some limitations. First, although we were able to collect data from 188 individual animals, some groups had small sample size. Additionally, only 4 samples per sex per strain were evaluated for lacunar (XRM) and bone composition (Raman spectroscopy) analysis. The interpretation of these outcomes, especially those with high variability within a group should be supported by more data. Similarly, only 25 DO animals per sex were sampled, and adding more DO animals might alter some findings reported here. Second, because 2 of the inbred strains (NOD, NZO) are commonly used as models for diabetes, we tracked the glycemic status of all mice. Two groups consistently became hyperglycemic (NOD females, NZO males). Yet in NOD and NZO mice, most traits did not exhibit a large sex difference even though hyperglycemia only occurred in one sex, which suggests that these traits were not strongly affected by hyperglycemia in our study. Third, the multiscale traits were not captured from a single skeletal site (bone), which was due in part to the destructive nature of some assays. For this reason, we do not present correlations of traits collected from different bones. Finally, we only focused on cortical bone and did not investigate differences in cancellous bone within the 2 populations. Because of the importance of cancellous microarchitecture for bone health and osteoporotic fracture risk, future studies are warranted to investigate variations in cancellous bone traits in these populations of mice.

In summary, we find that multi-scale cortical bone traits vary with genetic background and are moderately to highly heritable. Although we note significant variations in bone composition, these were less pronounced than variations in bone morphology, indicating that the bone composition between diverse mice is well conserved, whereas bone distribution varies widely. This study has several novel findings. First, lacunar morphology traits are genetically regulated. Second, we directly compared bone phenotypes between the DO mice and their inbred progenitors (inbred founders); this provides insight into how traits change with outbreeding. Lastly, we demonstrate conservation of intrabone relationships in multiple genetically diverse populations, providing support for how robust these relationships are for *M. musculus*. This work supports future use of these genetically diverse populations to discover novel genes contributing to cortical bone traits, including at the lacunar length scale.

## Supplementary Material

Captions_Supplemental_Figures_and_Tables_submit_ziae019

## Data Availability

All raw data is available in the Supplementary excel files uploaded online with this manuscript.
